# Genomic Characterization of Linezolid-Resistant *Clostridioides difficile* Harboring *cfr* Variants

**DOI:** 10.3390/biotech14020042

**Published:** 2025-05-31

**Authors:** Aikaterini Panou, Andigoni Malousi, Melina Kachrimanidou

**Affiliations:** 1 Department of Microbiology, Medical School, Aristotle University of Thessaloniki, 54124 Thessaloniki, Greece; 2 Laboratory of Biological Chemistry, Medical School, Aristotle University of Thessaloniki, 54124 Thessaloniki, Greece

**Keywords:** *C. difficile*, *Cfr* subtypes, ST, linezolid, antimicrobial resistance genes

## Abstract

The emergence of antimicrobial resistance (AMR) in *Clostridium difficile* (*C. difficile*), particularly to last-line antibiotics such as linezolid, represents a critical challenge in clinical settings. This study investigates the genomic epidemiology of linezolid-resistant *C. difficile*, focusing on the distribution and mutational patterns of the chloramphenicol–florfenicol resistance (*cfr*) gene and its association with multidrug resistance. We analyzed 514 clinical isolates (354 from NCBI Pathogen Detection, 160 from EnteroBase), revealing distinct prevalence patterns among *cfr* subtypes: *cfr*(C) was dominant (156/354 NCBI strains; 101/160 EnteroBase strains), whereas *cfr*(B) frequently harbored missense mutations (p.R247K, p.V294I, and less commonly p.A334T). The *cfr*(E) subtype was exclusively identified in ribotype 027 (RT027) strains. Notably, *cfr*(C) exhibited a strong association with RT017, correlating with a conserved 99 bp genomic deletion. Phylogenetic analysis linked *cfr*-carriage to predominant sequence types (ST1 in NCBI strains, ST37 in EnteroBase isolates). Furthermore, the co-occurrence of *cfr* with additional AMR genes conferred resistance to macrolides (erythromycin, azithromycin) and tetracyclines, indicating a convergent evolution toward multidrug resistance. These findings underscore the interplay between *cfr* mutations, hypervirulent ribotypes, and AMR dissemination, necessitating enhanced surveillance to mitigate the spread of resistant *C. difficile* lineages.

## 1. Introduction

*Clostridioides difficile* (formerly *Clostridium difficile*, *C. difficile*) is a Gram-positive, spore-forming, anaerobic bacillus that colonizes the gastrointestinal tract of humans and produces toxins [1]. It is the primary cause of infectious diarrhea in hospitalized patients, with broad-spectrum antibiotics being the major risk factor, as they disrupt gut microbiota, facilitating spore germination and toxin production. The pathogenesis of this microorganism is mainly driven by two exotoxins, toxin A and toxin B, which are encoded in the Pathogenicity Locus (PaLoc) of the *C. difficile* genome [2,3]. The *C. difficile* genome comprises 622–3000 genes and ranges in size from 4.1 to 4.3 Mbp, depending on the strain [4]. Certain *C. difficile* strains produce a third toxin, known as binary toxin (CDT), an ADP-ribosyltransferase that is commonly found in strains with increased severity of *C. difficile* infection [5].

*C. difficile* infection presents with a range of clinical symptoms, from mild diarrhea to severe and potentially fatal pseudomembranous colitis and toxic megacolon [6]. Historically, CDI was mainly considered a healthcare-associated infection (HA-CDI). In recent years, the occurrence, severity, recurrence rates, and mortality tied to CDI have markedly risen. The latest surveillance data from the CDC indicate that the overall crude incidence rate is 116.1 cases per 100,000 individuals [7], showing a greater incidence of community-associated cases (62.1 cases per 100,000 persons) than healthcare-associated cases (54.0 cases per 100,000) [8]. In addition, notable changes in the worldwide epidemiology of the disease have been noted, with a considerable increase in the prevalence of CDI outside the hospital setting being observed [9]. The accumulation of antimicrobial resistance in *C. difficile* may increase the risk of CDI occurrence and its transmission. The restricted availability of antimicrobials for treating CDI raises some concerns [10]. Antibiotic resistance significantly influences the epidemiology of *C. difficile.* The emergence of new hypervirulent strains is frequently linked to the rise of new resistance, and most epidemic *C. difficile* clinical isolates are resistant to multiple antibiotics. It was demonstrated that limiting the use of fluoroquinolones, clindamycin, amoxicillin/clavulanate, and cephalosporins was an effective antibiotic stewardship strategy for reducing the prevalence of multidrug-resistant epidemic ribotypes such as 001 and 027 [11,12]. For the treatment of CDI, three antimicrobial agents are currently recommended: metronidazole, vancomycin, and fidaxomicin [13,14]. Resistance to metronidazole, vancomycin, fidaxomicin, meropenem, and piperacillin/tazobactam is rarely reported.

Resistance to linezolid has been reported in some clinical isolates of *C. difficile* [15,16]. While linezolid-resistant *C. difficile* strains are relatively rare, their emergence and spread could potentially lead to a rise in *C. difficile* infections, particularly in healthcare settings where linezolid is frequently used.

In this study, we focus on strains of *C. difficile* that are probably resistant to linezolid. Linezolid is the first antibiotic in the oxazolidinone group and is identified as a last-line antibiotic, along with vancomycin and daptomycin. These antibiotics are used for the treatment of multidrug-resistant pathogens. Linezolid inhibits the synthesis of proteins in bacteria through its binding to 30S and 50S ribosomal subunits of rRNA. As a result, the initiation complex cannot be formed, and the length of developed peptide chains will be decreased. Furthermore, linezolid may reduce the amount of toxins that Gram-positive pathogens produce. The FDA (Food and Drug Administration) has approved linezolid for the treatment of infections caused by *Staphylococcus aureus*, either methicillin-susceptible or methicillin-resistant (MSSA, MRSA); *Streptococcus pneumoniae*; vancomycin-resistant *Enterococcus faecium* (VREF); *Streptococcus pyogenes*; and *Streptococcus agalactiae* [17].

Resistance to linezolid in *Enterococcus* spp. and *Staphylococcus* spp. is linked to the presence of the *cfr*, *optrA*, and *poxtA* genes [18]. In *C. difficile* strains, the *clcD* gene, also known as *cfr*(B), appears to contribute to linezolid resistance [19]. The *cfr* gene encodes a radical S-adenosyl-L-methionine (SAM) enzyme that promotes cross-resistance to antibiotics targeting the 23S rRNA by hypermethylating nucleotide A2503. The *Cfr* gene is typically found within mobile genetic elements, which highlights its genetic variability and suggests its role in the expansion of resistance mechanisms. In *C. difficile* strains, three subtypes of *cfr*—*cfr*(B), *cfr*(C), and *cfr*(E)—have been identified and are linked to resistance against antibiotics such as linezolid [20]. Furthermore, a recent study associates linezolid resistance in *C. difficile* strains with the presence of the *cfr* gene [21].

This study examines the genomic profile of linezolid-resistant *C. difficile* clinical isolates available in the online databases NCBI and EnteroBase. We aim to establish a genomic profile of these linezolid-resistant strains by analyzing their sequence types (STs) and ribotypes (RTs) to gain insight into the molecular basis of their resistance and to correlate the presence of specific subtypes of the *cfr* gene with hypervirulent strains.

## 2. Materials and Methods

### 2.1. Data Collection

Data were collected from two genomic resources, the National Center for Biotechnology Information (NCBI) Pathogen Detection and the EnteroBase (v1.2.0) [22]. Epidemiological data for *C. difficile* were retrieved from NCBI, with a focus on isolates harboring *cfr* gene subtypes to construct a linezolid-resistant strain collection. The selected strains possessed the *cfr* gene and exhibited linezolid resistance, consistent with the predicted phenotypic profiles based on the provided dataset. EnteroBase was additionally utilized to obtain metadata on PCR ribotypes and human-source niche information. This information was used as a selection criterion within the database.

### 2.2. Data Analysis

Isolates meeting the selection criteria were further annotated to include multilocus sequence typing (MLST) data (v2.23.0) [23] using the Staramr tool (v0.10.0) [24], which integrates antimicrobial resistance profiling via ResFinder (v4.6.0) [25] and plasmid detection via PlasmidFinder (v2.1) [26]. Strains lacking an assigned ST, according to the Staramr tool, were excluded from subsequent analyses.

To build distinct genomic profiles of linezolid-resistant strains, we retrieved an equivalent number of randomly selected *C. difficile* strains lacking AMR genes from NCBI’s Pathogen Detection resource, attempting to adhere to the geographical distribution pattern of the linezolid-resistant strains. The statistical significance of ST distributions was assessed using chi-square tests to estimate the *p*-value. The frequency of every ST was compared to each other on chi-square in order to estimate the *p*-value; *p*-value < 0.05 indicates statistical importance.

Genome annotation was performed using Prokka (Version 1.14.6) [27] and the default “Bacteria” database setting, while multiple sequence alignments of antimicrobial resistance genes were constructed using the Clustal Omega (version 1.2.4) [28] and CARD (v3.2.5) database records. The strain used as the reference for the *cfr*(*C*) mutation is defined as Ribosomal RNA large subunit methyltransferase *cfr* (*Clostridioides difficile* T10), RT126, registered in GenBank under accession number CCL89685. All analyses were conducted programmatically in R/RStudio (v4.3.2), with phylogenetic trees constructed using the distance-based clustering method of Clustal Omega with default parameters and visualized using the iTOL platform (v7) [29]. Additionally, the Sequence Manipulation Suite [30] was employed to translate gene sequences into proteins as part of the mutational analysis, and Jalview version 2.11.4.1 [31] was used to comparatively visualize multiple sequence alignments and to visually locate polymorphic loci across the *cfr* gene bodies.

## 3. Results

### 3.1. Description of Linezolid-Resistant C. difficile Strains

**Figure 1:**
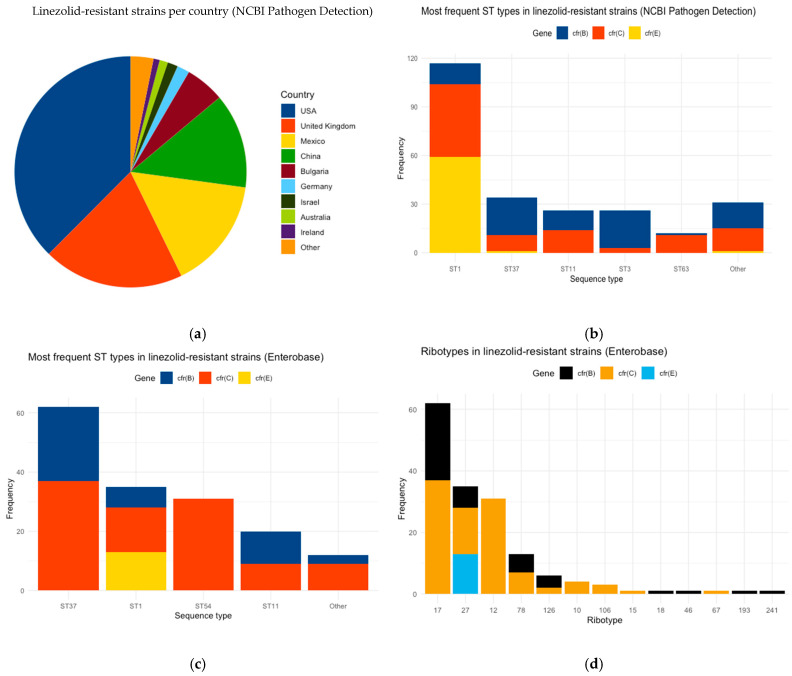
Linezolid-resistant *C. difficile* strains: (**a**) country of isolation for linezolid-resistant strains of NCBI Pathogen Detection; (**b**) distribution of ST types and *cfr* gene in linezolid-resistant strains of NCBI Pathogen Detection; (**c**) distribution of STs and *cfr* gene in linezolid-resistant strains of EnteroBase; (**d**) distribution of PCR ribotypes and *cfr* gene in linezolid-resistant strains of EnteroBase.

NCBI’s Pathogen Detection database included 27,248 *C. difficile* strains (retrieved 25 July 2024), of which 354 were clinical isolates, including *cfr*(*B*), *cfr*(*C*), *and cfr*(*E*) subtypes, covering the period between 2017 and 2024. The *cfr*(*C*) subtype was the most prevalent, identified in 156 strains, followed by *cfr*(*B*) (117 strains) and *cfr*(*E*) (81 strains). Geographically, 35% of the isolates originated from the USA, 18% from the United Kingdom, 14% from Mexico, and 11% from China (Figure 1a). ST1 was the most frequently observed ST, counting 117 strains, and includes all subtypes: 13 strains with *cfr*(B), 45 with *cfr*(C), and 59 with *cfr*(E) (Figure 1b). ST37, ST11, ST3, and ST63 were detected in fewer strains (34, 26, 26, and 12, respectively), while 110 out of 354 NCBI strains did not have an assigned ST. Furthermore, *cfr*(E)-including strains mainly belonged to ST1, while strains with the *cfr*(B) and *cfr*(C) subtypes were assigned to various STs.

The EnteroBase dataset comprised 2498 clinical *C. difficile* strains isolated from PCR ribotype-annotated human samples (retrieved 6 November 2024). The isolates deposited in EnteroBase were collected over the period between 1991 and 2022. Of these, 160 were linezolid-resistant, including all three *cfr* gene subtypes. The *cfr*(C) subtype was identified in 101 strains, 46 strains included the *cfr*(B) subtype, and 13 the *cfr*(E) subtype. Of the 160 strains, 136 were also included in the NCBI database. Additionally, 53 strains were associated with hypervirulent RTs (35 RT027, 5 RT126, 13 RT78). The most common STs among linezolid-resistant strains were 62 ST37, 35 ST1, 31 ST54, and 20 ST11 (Figure 1c), while two strains were not assigned to any ST. In accordance with the analysis of the NCBI strains, the *cfr*(E) gene was found exclusively in ST1. The ST37 and ST11 strains included both *cfr*(B) and *cfr*(C) subtypes, while 31 strains of ST54 included only the *cfr*(C) subtype (Figure 1c). Regarding PCR ribotyping, 39% of the strains belonged to RT17, 22% to RT27, and 19% to RT12. Notably, the *cfr*(E) subtype was identified exclusively in the hypervirulent RT27 strains. The RT12, RT10, RT106, RT15, and RT67 strains were only associated with the *cfr*(C) subtype. Thirty-one RT12 strains had *cfr*(C), while the other RTs were found in lower percentages. Additionally, each RT18, RT46, RT193, and RT241 strain had only *cfr*(B) (Figure 1d).

### 3.2. Comparison Between Linezolid-Resistant and Susceptible C. difficile Strains of NCBI

**Figure 2:**
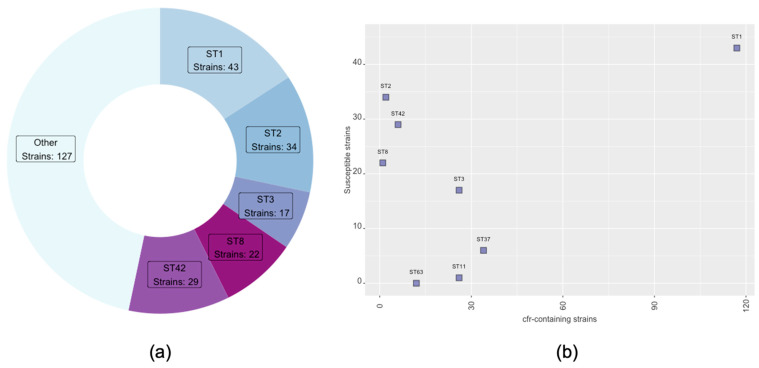
(**a**) Most frequent STs in susceptible *C. difficile* strains. (**b**) Distribution of different STs in strains with *cfr* gene (x axis) and in strains lacking AMR genes (y axis).

To evaluate the potential association of ST and RT profiles with linezolid resistance, we sought to compare them with susceptible *C. difficile* strains. Thus, 354 randomly selected *C. difficile* clinical strains lacking AMR genes were chosen from NCBI and compared with an equal number of linezolid-resistant strains from the NCBI dataset. Of the 354 strains, 82 strains were not assigned to a specific ST, and among the remaining 272 strains, ST1 accounted for 43 strains, ST2 for 34 strains, ST42 for 29, ST3 for 17, and ST8 for 22 strains (Figure 2a). The remaining 47% (127 strains) included strains that had a variety of different STs with lower frequency, ranging from 1% to 3%. In addition, most of them were isolated in the USA (56.7%). Figure 2b shows the distribution of the STs across the linezolid-resistant strains and the sensitive strains. A clear distinction is observed in STs associated with sensitive and linezolid-resistant strains. ST2, ST8, and ST42 are mainly linked to sensitive strains lacking antimicrobial AMR genes, whereas ST37, ST11, and ST63 are primarily associated with linezolid-resistant strains. The *p*-values of ST1, ST2, ST8, ST42, ST11, ST37, and ST63 are statistically significant (*p*-value < 0.00001), while for ST3, no statistically significant differences are observed (*p*-value = 1). ST1 is the most abundant in both categories (Figure 2b).

### 3.3. Mutation Analysis of EnteroBase C. difficile Strains

To assess the differences in the mutation spectrum of *cfr* subtypes, we applied multiple alignments of each *cfr* subtype based on the assigned RTs and STs. We analyzed the *cfr*(B) and *cfr*(C) sequences since their strains exhibit a diverse range of RTs and STs. *cfr*(E) was not examined because all *cfr*(E) strains belong to RT27 (ST1).

**Figure 3:**
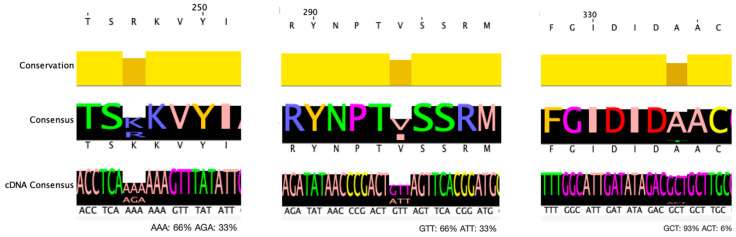
Polymorphic loci and conservation level of *cfr*(B) gene. From left to right, mutant amino acids p.R247K, p.V294I, and p.A334T are shown, and the corresponding nucleotide substitutions.

Of the 46 *cfr*(B)-containing strains, 44 matched the expected 1050 bp length, while the remaining 2 were truncated at the 3′ end of the gene (738 bp, 981 bp). The comparison of the 44 gene sequences with the *cfr*(B) gene of the *C. difficile* obtained by the CARD database (gb|HG002396.1) revealed the presence of two G>A missense mutations at positions 740 and 880 corresponding to amino acid mutations p.R247K and p.V294I, respectively. A third G>A missense mutation at position 1000 corresponding to p.A334T is also observed in three strains, all also carrying both p.R247K and p.V294I mutations. The frequency of the mutant strains per ST is shown in Table 1. The polymorphic loci of *cfr*(B) gene are presented in Figure 3.

There were four specific STs for the mutant subtypes ST11, ST1, ST17, and ST37. ST37 is present in all the subtypes carrying the mutation, while ST11 is the most abundant in genes with mutations p.R247K and p.V294I. Every strain possessing the p.R247K mutation also has the p.V294I mutation; however, not all strains with the p.V294I mutation have the p.R247K mutation. Furthermore, all seven RT27 strains containing *cfr*(B) also had the p.V294I mutation.

Of the 44 strains with *cfr*(B), 16 were associated with hypervirulent RTs (RT17, RT126, RT78). The p.R247K mutation is present in half of the hypervirulent strains. The p.V294I mutation was linked to all 16 hypervirulent strains with the *cfr*(B) subtype.

Furthermore, considering *cfr*(C), there were 103 strains carrying the *cfr*(C) subtype, of which 99 had either 1251 bp or 1152 bp. Mutational analysis was performed solely on the variants detected in these 99 samples. Twenty-two RT17 (ST37) strains with 1152 bp were distinguished by a 99 bp sequence deletion at the 5′ end of the gene. Interestingly, all four RT10 (ST15) strains had a polymorphic site at position 280 (g.280G>A, p.E94K) according to the reference sequence with accession number CCL89685 [20]. In addition, there are sporadically identified mutations that are found in a limited number of strains. There are also observed missense mutations in RT27 (ST1) strains: three strains with p.C111Y, four with p.I134N, and three strains with p.C142Y. Notably, three (out of four) strains carrying the p.I134N mutation also harbored the p.C142Y mutation, indicating that these three strains contain both mutations.

### 3.4. Phylogenetic Analysis

**Figure 4:**
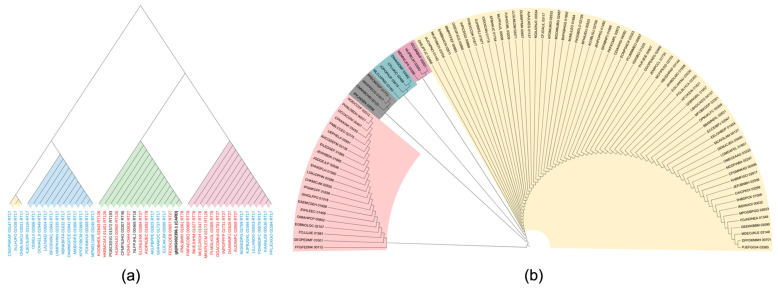
(**a**) Phylogenetic tree of 44 *cfr*(B) gene: red: gene subtypes of strains with RT27, RT78, RT126; black and blue: gene subtypes of strains with no hypervirulent STs. Green clade includes strains with p.R247K and p.V294I mutations, pink clade includes strains with p.V294I mutation and yellow clade includes strains with p.A334T mutation. Blue clade includes the rest of the strains. (**b**) Phylogenetic tree of 99 *cfr*(C) gene. The gene subtypes with RT17 are colored pink, the gene subtypes with the p.C111Y mutation are purple, the gene subtypes with the p.I134N and p.C142Y mutations are grey, and the gene subtypes with no important difference in alignment are colored yellow.

The phylogenetic analysis of the 44 *C. difficile* strains carrying *the cfr*(B) gene from EnteroBase is shown in Figure 4a. The red-marked *cfr*(B) subtype corresponds to hypervirulent strains, such as RT27, RT126, and RT78. Hypervirulent strains are found in two distinct clades, both originating from the same source. The strains that are represented in the green clade are those that have both the p.R247K and p.V294I mutations, while the strains that belong to the pink clade are those that have only the p.V294I mutation. The yellow clade includes all p.A334T mutant strains.

Figure 4b shows the phylogenetic tree of the 99 strains with the *cfr*(C) subtype from EnteroBase. The clade with pink color includes the strains that have the 99 bp deletion part at the 5′edge and differ from the rest of the subtypes the most. The strains that are marked in blue are the four RT17 (ST37) that have the p.E94K mutation. The purple clade includes strains that have the p.C111Y mutation, and grey branches include strains with the p.I134N and p.C142Y mutations, except the strain of the CMNKBCHN_02120 gene, which is the only one that exclusively has the p.I134N mutation. The rest of the yellow clade has no important phylogenetic differences.

### 3.5. Presence of Other AMR Genes

Among 354 linezolid-resistant strains included in the NCBI’s Pathogen Detection resource, 249 of the strains from the NCBI database have at least one more AMR gene in their genome.

The most abundant gene that coexists with *cfr* is the *erm* gene, which is associated with resistance to erythromycin and azithromycin. There are 195 strains with *erm*(A), *erm*(B), and *erm*(*Q*) subtypes. A total of 180 strains have *erm*(B), of which 68 strains include the combinations *cfr*(E)-*erm*(B), 69 *cfr*(C)-*erm*(B), and 43 *cfr*(B)-*erm*(B) subtypes. 

Furthermore, *tet* genes that are associated with resistance to tetracycline are present in 76 strains with *cfr* subtypes, 66 strains with *tet*(M), and 10 with *tet*(*32*) only in combination with *cfr*(B). There are 60 strains with the *aac*(*6*′)-*aph*(*2*″) genes that are associated with resistance to amikacin, gentamicin, and tobramycin, and 77 strains with the *ant*(*6*)-*Ia* gene with resistance to streptomycin are also detected. (15 *aph*(*3*′)-*III*, which is associated with resistance to kanamycin and amikacin, and 7 *aph*(*2*″)-*If*, which is associated with resistance to amikacin, gentamicin, and tobramycin).

Interestingly, besides the *tet* gene, we found other AMR genes that coexist only with certain *cfr* subtypes, such as (a) 8 strains include *fos*B3 (which provides resistance to fosfomycin), which coexist only with *cfr*(E) and *erm*(B); (b) 16 strains include *catP* (resistance to chloramphenicol), which is combined only with *cfr*(B); and (c) 3 strains include *qnrB19* (resistance to ciprofloxacin I/R), which is combined only with *cfr*(E) (Table 2).

## 4. Discussion

In this study, we aimed to analyze linezolid-resistant *C. difficile* clinical isolates available in the online databases NCBI Pathogen Detection and EnteroBase. We specifically aimed to establish a genomic profile of linezolid-resistant *C. difficile* strains by analyzing their STs, RTs, and mutational spectrum to gain insights into the molecular basis of their resistance and to correlate the presence of specific *cfr* subtypes with hypervirulent strains.

The distribution of *cfr* gene subtypes across publicly available databases reveals significant trends in their prevalence. Our analysis indicates that *cfr*(*C*) is the most common subtype, being present in 156 out of 354 strains in NCBI and 101 out of 160 strains in EnteroBase.

As regards the ST distribution, ST1 and ST37 are the most abundant STs in both datasets. This finding aligns with expectations, given that some strains cataloged in EnteroBase are also included in NCBI, leading to an overlap in the dataset composition. Moreover, a notable association is observed between *cfr*(*E*) and ST1 across both datasets, as has been described in previous studies [32,33]. Interestingly, *cfr*(*E*) is exclusively found in strains with RT27, indicating a potential linkage between *cfr*(*E*) and specific strain lineages. Furthermore, it is observed that RT17 is the predominant ribotype in EnteroBase, recognized for its extensive prevalence [33]. Moreover, ST54 is found to be exclusively associated with *cfr*(*C*), a correlation that has not been previously reported.

Further comparative analysis of NCBI’s strains carrying the *cfr* gene against an equal number of randomly selected strains devoid of AMR genes confirms that ST1 is the most frequent sequence type. Interestingly, it is observed that there is a pattern in STs; susceptible strains are mainly associated with ST2, ST8, and ST42, while linezolid-resistant strains are mainly included in ST37, ST11, and ST63.

The genomic sequence comparison of *cfr* subtypes revealed critical genetic variations. Specifically, in the *cfr*(*B*) gene, two A>G variant loci were identified at positions 740 and 880, which have not been referred to in any other report previously. These missense mutations are p.R247K and p.V294I, respectively, in the producing protein. All strains possessing the p.R247K mutation also have the mutation p.V294I. The missense mutation p.R247K replaces the amino acid lysine (K) with arginine (R), and the missense mutation p.V294I replaces the amino acid valine (V) with isoleucine (I) in the protein. These strains belong to four distinct STs: ST1, ST11, ST37, and ST3. Such alterations may have functional implications in the activity or stability of the *cfr*(*B*) protein. The third p.A334T mutation is also present in strains that have the p.R247K and p.V294I mutations, which probably affects the virulence of these strains.

The alignment of the *cfr*(*C*) gene highlights 22 RT17 (ST37) strains with a 99 bp deletion on the 5′ end of the gene. The rest of the mutations are missense and replace cysteine with tyrosine in p.C111Y, isoleucine with asparagine in p.I134N, and cysteine with tyrosine in p.C142Y. All these strains are RT27 (ST1), which is a hypervirulent RT. The limited occurrence of these mutations suggests a potential niche adaptation or selective pressure within these ribotypes.

A critical observation of our study is the frequent coexistence of the *cfr* gene with other AMR genes, such as *erm*, *tet*, *aac*(*6*′)-*aph*(*2*″), *ant*(*6*)-*Ia*, *fosB3*, *catP*, and *qnrB19*. This co-resistance pattern suggests that *C. difficile* strains carrying the *cfr* gene are often multidrug-resistant, including linezolid, erythromycin, azithromycin, tetracycline, amikacin, gentamicin, tobramycin, streptomycin, fosfomycin, chloramphenicol, and ciprofloxacin. The presence of such resistance determinants highlights the clinical challenge posed by these strains and underscores the necessity for continuous surveillance and alternative therapeutic strategies [20].

## 5. Limitations of the Study

We acknowledge limitations in our study, particularly that clinical data were unavailable for *cfr*-carrying *C. difficile* isolates, restricting our ability to assess the clinical relevance of the observed mutations. In addition, the strains retrieved from the datasets were predominantly isolated in the USA, suggesting that the observed distribution may be biased due to the geographic limitations of the available data.

## 6. Conclusions

Overall, analyzing hundreds of *C. difficile* strains offers robust insight into genetic variation by profiling mutations and investigating their relationship to antimicrobial resistance markers. Our findings contribute to a deeper understanding of the *cfr* gene distribution in *C. difficile* strains. It is observed that several mutations of the *cfr* gene are associated with hypervirulent and non-hypervirulent RTs. The identified genetic correlations and patterns of co-resistance highlight the essential role of ongoing surveillance to inform and guide successful antimicrobial stewardship strategies and effective therapeutic agents.

## Figures and Tables

**Table 1:** STs and RTs of mutant *cfr*(B) gene.

Amino Acid Mutations of *cfr*(B) Gene	Number of Strains	ST11	ST1	ST17	ST37	no ST
p.R247K	14/44	6 (RT78, RT126, RT193)	3 (RT27)	1 (RT18)	3 (RT17)	1 (RT46)
p.V294I	29/44	11 (RT78, RT126, RT193)	7 (RT27)	1 (RT18)	9 (RT17)	1 (RT46)
p.R247K & p.V294I	14/44	6 (RT78, RT126, RT193)	3 (RT27)	1 (RT18)	3 (RT17)	1 (RT46)
p.A334T	3/44	-	-	-	3 (RT17)	-
All three mutations	3/44	-	-	-	3 (RT17)	-

**Table 2:** Coexistence of AMR genes with *cfr* in 249 NCBI strains.

AMR Gene	Antibiotic of Resistance	Coexistence with *cfr* Gene			No. of Strains per Gene
		*cfr*(B)	*cfr*(c)	*cfr*(E)	
*erm*(B)	Erythromycin, Azithromycin	43	69	68	195
*erm*(Q)	Erythromycin, Azithromycin	2	2	-	
*erm*(A)	Erythromycin, Azithromycin	10	1	-	
*tet*(m)	Tetracycline	66	-	-	76
*tet*(32)	Tetracycline	10	-	-	
*aac*(*6*′)-*aph*(*2*″)	Amikacin, Gentamicin, Tobramycin	42	12	6	60
*ant*(*6*)-*Ia*	Streptomycin	41	35	1	77
* fosB3 *	Fosfomycin	-	-	8	8
* catP *	Chloramphenicol	16	-	-	16
* qnrB19 *	Ciprofloxacin I/R	-	-	3	3

## Data Availability

No new data were created or analyzed in this study.

## References

[1] Smits W.K., Lyras D., Lacy D.B., Wilcox M.H., Kuijper E.J. (2016). *Clostridium Difficile* Infection. Nat. Rev. Dis. Primers.

[2] Di Bella S., Ascenzi P., Siarakas S., Petrosillo N., di Masi A. (2016). *Clostridium Difficile* Toxins A and B: Insights into Pathogenic Properties and Extraintestinal Effects. Toxins.

[3] Sehgal K., Khanna S. (2021). Gut Microbiome and *Clostridioides Difficile* Infection: A Closer Look at the Microscopic Interface. Ther. Adv. Gastroenterol..

[4] Wang J., Yang C., Zhang C., Mao X., Lizhe A. (2021). Complete Genome Sequence of the *Clostridium Difficile* LCL126. Bioengineered.

[5] Gerding D.N., Johnson S., Rupnik M., Aktories K. (2013). *Clostridium Difficile* Binary Toxin CDT: Mechanism, Epidemiology, and Potential Clinical Importance. Gut Microbes.

[6] Czepiel J., Dróżdż M., Pituch H., Kuijper E.J., Perucki W., Mielimonka A., Goldman S., Wultańska D., Garlicki A., Biesiada G. (2019). *Clostridium Difficile* Infection: Review. Eur. J. Clin. Microbiol. Infect. Dis..

[7] Vindigni S.M., Surawicz C.M.C. (2015). *C. Difficile* Infection: Changing Epidemiology and Management Paradigms. Clin. Transl. Gastroenterol..

[8] Centers for Disease Control and Prevention (2022). Centers for Disease Control and Prevention. Emerging Infections Program, Healthcare-Associated Infections—Community Interface Surveillance Report, Clostridioides Difficile Infection (CDI).

[9] Khanna S., Pardi D.S., Aronson S.L., Kammer P.P., Orenstein R., St Sauver J.L., Harmsen W.S., Zinsmeister A.R. (2012). The Epidemiology of Community-Acquired *Clostridium Difficile* Infection: A Population-Based Study. Am. J. Gastroenterol..

[10] Rupnik M., Wilcox M.H., Gerding D.N. (2009). *Clostridium Difficile* Infection: New Developments in Epidemiology and Pathogenesis. Nat. Rev. Microbiol..

[11] Spigaglia P., Mastrantonio P., Barbanti F. (2018). Antibiotic Resistances of *Clostridium Difficile*. Adv. Exp. Med. Biol..

[12] Lawes T., Lopez-Lozano J.M., Nebot C.A., Macartney G., Subbarao-Sharma R., Wares K.D., Sinclair C., Gould I.M. (2017). Effect of a National 4C Antibiotic Stewardship Intervention on the Clinical and Molecular Epidemiology of *Clostridium Difficile* Infections in a Region of Scotland: A Non-Linear Time-Series Analysis. Lancet Infect. Dis..

[13] McDonald L.C., Gerding D.N., Johnson S., Bakken J.S., Carroll K.C., Coffin S.E., Dubberke E.R., Garey K.W., Gould C.V., Kelly C. (2018). Clinical Practice Guidelines for *Clostridium Difficile* Infection in Adults and Children: 2017 Update by the Infectious Diseases Society of America (IDSA) and Society for Healthcare Epidemiology of America (SHEA). Clin. Infect. Dis..

[14] Debast S.B., Bauer M.P., Kuijper E.J., Allerberger F., Bouza E., Coia J.E., Cornely O.A., Fitzpatrick F., Guery B., Wilcox M. (2014). European Society of Clinical Microbiology and Infectious Diseases: Update of the Treatment Guidance Document for *Clostridium Difficile* Infection. Clin. Microbiol. Infect..

[15] Alcalá L., Martín A., Marín M., Sánchez-Somolinos M., Catalán P., Peláez T., Bouza E., Spanish *Clostridium difficile* Study Group (2012). The Undiagnosed Cases of *Clostridium Difficile* Infection in a Whole Nation: Where Is the Problem?. Clin. Microbiol. Infect..

[16] Spigaglia P., Barbanti F., Mastrantonio P., Ackermann G., Balmelli C., Barbut F., Bouza E., Brazier J., Delmée M., Drudy D. (2011). Multidrug Resistance in European *Clostridium Difficile* Clinical Isolates. J. Antimicrob. Chemother..

[17] Hashemian S.M.R., Farhadi T., Ganjparvar M. (2018). Linezolid: A Review of Its Properties, Function, and Use in Critical Care. Drug Des. Dev. Ther..

[18] Kowalewicz C., Timmermans M., Fretin D., Wattiau P., Boland C. (2023). An In-House 45-Plex Array for the Detection of Antimicrobial Resistance Genes in Gram-Positive Bacteria. MicrobiologyOpen.

[19] Candela T., Marvaud J.C., Nguyen T.K., Lambert T. (2017). A *Cfr*-like Gene *Cfr*(C) Conferring Linezolid Resistance Is Common in *Clostridium Difficile*. Int. J. Antimicrob. Agents.

[20] Stojković V., Ulate M.F., Hidalgo-Villeda F., Aguilar E., Monge-Cascante C., Pizarro-Guajardo M., Tsai K., Tzoc E., Camorlinga M., Paredes-Sabja D. (2020). *Cfr*(B), *Cfr*(C), and a New *Cfr*-Like Gene, *Cfr*(E), in *Clostridium Difficile* Strains Recovered across Latin America. Antimicrob. Agents Chemother..

[21] Plankaova A., Brajerova M., Capek V., Balikova Novotna G., Kinross P., Skalova J., Soltesova A., Drevinek P., Krutova M. (2023). *Clostridioides Difficile* Infections Were Predominantly Driven by Fluoroquinolone-Resistant *Clostridioides Difficile* Ribotypes 176 and 001 in Slovakia in 2018–2019. Int. J. Antimicrob. Agents.

[22] Zhou Z., Alikhan N.-F., Mohamed K., Fan Y., Achtman M. (2020). The EnteroBase User’s Guide, with Case Studies on *Salmonella* Transmissions, *Yersinia Pestis* Phylogeny, and *Escherichia* Core Genomic Diversity. Genome Res..

[23] Larsen M.V., Cosentino S., Rasmussen S., Friis C., Hasman H., Marvig R.L., Jelsbak L., Sicheritz-Pontén T., Ussery D.W., Aarestrup F.M. (2012). Multilocus Sequence Typing of Total-Genome-Sequenced Bacteria. J. Clin. Microbiol..

[24] Bharat A., Petkau A., Avery B.P., Chen J.C., Folster J.P., Carson C.A., Kearney A., Nadon C., Mabon P., Thiessen J. (2022). Correlation between Phenotypic and In Silico Detection of Antimicrobial Resistance in Salmonella Enterica in Canada Using Staramr. Microorganisms.

[25] Bortolaia V., Kaas R.S., Ruppe E., Roberts M.C., Schwarz S., Cattoir V., Philippon A., Allesoe R.L., Rebelo A.R., Florensa A.F. (2020). ResFinder 4.0 for Predictions of Phenotypes from Genotypes. J. Antimicrob. Chemother..

[26] Carattoli A., Zankari E., García-Fernández A., Voldby Larsen M., Lund O., Villa L., Møller Aarestrup F., Hasman H. (2014). *In Silico* Detection and Typing of Plasmids Using PlasmidFinder and Plasmid Multilocus Sequence Typing. Antimicrob. Agents Chemother..

[27] Seemann T. (2014). Prokka: Rapid Prokaryotic Genome Annotation. Bioinformatics.

[28] Sievers F., Higgins D.G. Clustal Omega, Accurate Alignment of Very Large Numbers of Sequences. 2014, 1079, 105–116. Methods Mol. Biol..

[29] Letunic I., Bork P. (2024). Interactive Tree of Life (iTOL) v6: Recent Updates to the Phylogenetic Tree Display and Annotation Tool. Nucleic Acids Res..

[30] Stothard P. (2000). The Sequence Manipulation Suite: JavaScript Programs for Analyzing and Formatting Protein and DNA Sequences. BioTechniques.

[31] Waterhouse A.M., Procter J.B., Martin D.M.A., Clamp M., Barton G.J. (2009). Jalview Version 2—A Multiple Sequence Alignment Editor and Analysis Workbench. Bioinformatics.

[32] Chandrasekaran R., Lacy D.B. (2017). The Role of Toxins in *Clostridium Difficile* Infection. FEMS Microbiol. Rev..

[33] Aguilar-Zamora E., Weimer B.C., Torres R.C., Gómez-Delgado A., Ortiz-Olvera N., Aparicio-Ozores G., Barbero-Becerra V.J., Torres J., Camorlinga-Ponce M. (2022). Molecular Epidemiology and Antimicrobial Resistance of *Clostridioides Difficile* in Hospitalized Patients From Mexico. Front. Microbiol..

